# Development and Initial Psychometric Validation of the Multidimensional Menopausal Burden Questionnaire (MMBQ-44) in a Sample of Romanian Women

**DOI:** 10.3390/diagnostics16162574

**Published:** 2026-08-14

**Authors:** Ana-Cristiane Dragomir, Alina Ramona Buzatu, Ruxandra-Cristina Marin, Radu Dumitru Moleriu, Călin Muntean, Teodora Piroș, Iulia Najette Crintea, Andreea Anda Alexa, Anca Marcu, Marilena Dinuți

**Affiliations:** 1 Doctoral School, “Victor Babes” University of Medicine and Pharmacy, 300041 Timisoara, Romania; cristiane.dragomir@umft.ro; 2 Discipline of Biochemistry, Department of Biochemistry and Pharmacology, Faculty of Medicine, “Victor Babes” University of Medicine and Pharmacy, 2 Eftimie Murgu Square, 300041 Timisoara, Romania; buzatu.ramona@umft.ro (A.R.B.); alexa.anda@umft.ro (A.A.A.); marcu.anca@umft.ro (A.M.); dinuti.marilena@umft.ro (M.D.); 3 Discipline of Pharmacology, Clinical Pharmacology and Pharmacotherapy, “Carol Davila” University of Medicine and Pharmacy, 050474 Bucharest, Romania; 4 Doctoral School of Biological and Biomedical Sciences, University of Oradea, 410087 Oradea, Romania; 5 Department III, Functional Science, Discipline of Medical Informatics and Biostatistics, “Victor Babes” University of Medicine and Pharmacy, 300041 Timisoara, Romania; cmuntean@umft.ro (C.M.); teodora.piros@student.umft.ro (T.P.); 6 Department of Surgery I, Faculty of Medicine, “Victor Babes” University of Medicine and Pharmacy, 2 Eftimie Murgu Square, 300041 Timisoara, Romania; iulia.crintea@umft.ro; 7 Emergency Department, Emergency Clinical Municipal Hospital Timisoara, 300079 Timisoara, Romania

**Keywords:** menopause, perimenopause, questionnaire validation, psychometrics, xerostomia, oral health, estrogen deficiency, Menopause Rating Scale, Xerostomia Inventory, OHIP-14, exploratory factor analysis, Cronbach’s alpha, McDonald’s omega, HTMT

## Abstract

**Background/Objectives:** Current menopause assessment instruments primarily evaluate vasomotor, somatic, and urogenital symptoms, with limited attention to oral, sensory, and integrated psychological domains. Evidence that oral tissues are estrogen-responsive suggests that oral symptoms may represent an important but underrecognized component of menopausal burden. This study aimed to develop and provide initial psychometric validation of the Multidimensional Menopausal Burden Questionnaire (MMBQ-44), a 44-item instrument integrating systemic, oral, and psychological symptoms. **Methods:** A cross-sectional validation study was conducted in 308 women aged 45–65 years (perimenopausal *n* = 66, menopausal *n* = 40, postmenopausal *n* = 202). The MMBQ-44 combined items from the Menopause Rating Scale (MRS), Xerostomia Inventory (XI), Oral Health Impact Profile-14 (OHIP-14), and psychological measures derived from the PHQ-9, GAD-7, and HADS. Instrument development included expert review, cognitive interviewing, and field testing. Psychometric evaluation assessed item performance, internal consistency, factor structure, discriminant validity, and known-groups validity. Exploratory factor analysis used principal axis factoring with promax rotation, with factor retention determined by parallel analysis. **Results:** Internal consistency was good to excellent across domains (Cronbach’s α: 0.813–0.941; McDonald’s ω: 0.864–0.953). All corrected item–total correlations exceeded 0.30. The questionnaire showed strong factorability (KMO = 0.931; Bartlett’s χ^2^ = 9006.2, *p* < 0.001). Parallel analysis identified four factors explaining 48.6% of total variance. Psychological and OHIP domains emerged clearly, whereas several MRS and XI items cross-loaded onto related factors. Most HTMT ratios were below 0.85, supporting discriminant validity. Floor effects were observed for OHIP (37.0%) and XI (23.4%). Known-groups validity was modest, with OHIP scores differing significantly across menopausal stages. **Conclusions:** The MMBQ-44 demonstrates strong reliability and acceptable construct validity as an integrative screening instrument for menopausal burden. Further confirmatory studies and refinement of oral symptom items are warranted.

## 1. Introduction

Menopause is a physiological stage of reproductive aging characterized by progressive estrogen decline and the permanent cessation of ovarian follicular activity [1]. Contemporary clinical frameworks, including the STRAW+10 staging system, recognize menopause as a multidimensional transition associated with vasomotor, somatic, psychological, genitourinary, metabolic, and sleep-related manifestations [2,3] that may substantially impair quality of life [4,5,6].

In recent years, attention has increasingly shifted toward less traditionally assessed manifestations, particularly oral symptoms related to estrogen deficiency [7]. Hormonal changes may affect salivary secretion, oral mucosal integrity, periodontal tissues, and oral-sensory perception, contributing to xerostomia, burning mouth symptoms, taste disturbances, and reduced oral-health-related quality of life [8]. The theoretical framework underlying the present study is based on the concept that estrogen-sensitive epithelial and mucosal tissues may exhibit interconnected manifestations of systemic hormonal decline. Recent evidence indicates that oral tissues, similarly to vaginal mucosa, express estrogen receptors and may therefore undergo parallel inflammatory, trophic, sensory, and microbiological alterations during menopausal transition. Such manifestations are further shaped by neuroendocrine, immune, microbial and psychosocial interactions, and are therefore better understood as part of a broader multidimensional menopausal phenotype than as isolated dental findings [8,9].

Xerostomia represents one of the most clinically relevant oral manifestations associated with menopause because it may impair mastication, swallowing, speech, taste perception, sleep quality, and oral comfort [10]. However, dry-mouth symptoms in midlife women are multifactorial and may also be influenced by medication exposure, comorbidities, psychological distress, and aging-related salivary alterations [11]. Studies in postmenopausal women further support associations between menopausal status, oral dryness, and oral-health parameters [12].

Menopause-related oral alterations may also involve changes in the oral microbiome and mucosal environment, potentially increasing susceptibility to inflammatory and dysbiotic oral conditions [13,14]. Given the growing global postmenopausal population, these manifestations have increasing clinical relevance [15].

Oral manifestations of this kind—xerostomia, burning mouth symptoms, periodontal changes and altered taste perception—are common in midlife women and materially affect quality of life, yet remain insufficiently integrated into conventional menopause assessment tools [14,15,16,17,18,19,20].

Despite this evidence, menopausal assessment remains fragmented. Menopause-specific instruments such as the Menopause Rating Scale, the Menopause-Specific Quality of Life questionnaire, and the Greene Climacteric Scale are widely used to assess vasomotor, somatic, psychological, and sexual symptoms, but they do not provide a dedicated multidimensional evaluation of oral and oral-sensory burden [21].

Conversely, oral-health instruments such as the Xerostomia Inventory [22] and the Oral Health Impact Profile-14 [23] are valuable for evaluating dry-mouth symptoms and oral-health-related quality of life, but they are not menopause-specific and do not integrate systemic, urogenital, vasomotor, and psychological dimensions, and therefore cannot establish whether oral symptoms form part of a wider menopausal symptom cluster [24].

Psychological assessment is similarly compartmentalized. Generic tools such as the Patient Health Questionnaire-9 [25], Generalized Anxiety Disorder-7 scale [26] and Hospital Anxiety and Depression Scale [27] are well-established screening instruments, but they do not distinguish whether affective symptoms occur independently or in association with vasomotor symptoms, sleep disturbance, sexual discomfort, oral symptoms, or systemic menopausal burden. A domain-by-domain comparison of the MMBQ-44 with the menopause-specific, oral-health and psychological instruments currently in use is given in Supplementary Table S4; no existing instrument covers all four domains within a single administration.

This fragmentation may limit recognition of symptom clustering across systemic, oral, and psychological domains, even though these manifestations may share common endocrine, inflammatory, autonomic, and psychosocial mechanisms [28].

These limitations highlight the need for a concise multidimensional instrument capable of simultaneously assessing systemic, oral, and psychological aspects of menopausal burden within a unified framework [29]. The inclusion of oral manifestations is not intended to replace gynecological evaluation of estrogen-sensitive mucosae; the MMBQ-44 was designed to complement it, as an integrative screening and symptom-profiling instrument. This approach may be particularly relevant in community-based, primary-care, dental, or interdisciplinary settings where direct gynecological examination is not routinely performed, thereby supporting more integrated evaluation of menopausal burden.

No validated Romanian-language instrument currently assesses menopausal burden across systemic, oral and psychological domains: the instruments used in routine practice in Romania are single-domain, and none covers oral symptoms. Because gynecological, dental and mental-health services are organized as separate pathways with limited cross-referral, the oral component of a woman’s symptom burden is typically presented to a dental service and the systemic component to a gynecological service, with neither capturing the other. The research gap is therefore specific: no existing instrument permits the simultaneous, comparably scaled assessment of systemic, oral and psychological menopausal burden within a single administration, so symptom clustering across these domains cannot be examined without combining instruments of differing formats, response scales and recall periods.

In response to this unmet need for integrated assessment, we developed the Multidimensional Menopausal Burden Questionnaire (MMBQ-44), a novel questionnaire designed to capture four conceptually distinct but clinically interconnected dimensions of menopausal burden: systemic menopausal symptoms, xerostomia and oral-sensory symptoms, oral-health-related functional impact, and psychological burden. It was designed for routine administration, with an estimated completion time below 10 min.

The objectives of the present study were to describe the structured development of the MMBQ-44 through item generation, expert review, cognitive testing, and field administration; to provide initial psychometric evidence in a community-based sample of Romanian women aged 45–65 years; and finally, to evaluate internal consistency, item-level performance, factor structure, convergent and discriminant validity, known-groups validity, and floor and ceiling effects, with the aim of identifying domains or items requiring refinement before confirmatory validation in independent samples.

## 2. Materials and Methods

### 2.1. Study Design and Ethical Considerations

This was a single-centre, community-based, cross-sectional, questionnaire-based observational study conducted within the Department IV—Biochemistry and Pharmacology of the Victor Babeș University of Medicine and Pharmacy, Timișoara, Romania. The study was designed to evaluate multidimensional menopausal burden in community-dwelling midlife women and was not conducted within a dental clinic, oral-medicine service, or gynecological pathology referral setting.

Participant enrolment and questionnaire administration were performed between December 2025 and July 2026—that is, after ethical approval had been granted on 28 November 2025 (protocol Nr. 75/28.11.2025)—within the framework of a university-affiliated observational research project. Women were informed about the study through community-based voluntary recruitment and were invited to participate independently of oral-health status, dental-care attendance, or gynecological consultation.

All participants provided written informed consent prior to enrolment. Personal identifying data were not retained; each participant was assigned a unique numeric code in compliance with General Data Protection Regulation [30].

The study design and reporting followed the COSMIN methodology for studies on measurement properties [31] and were informed by the STARD reporting framework for diagnostic accuracy studies [32], where applicable.

### 2.2. Participants

A total of 308 community-based women aged 45–65 years were enrolled during the study period. The study was conducted using an online self-administered questionnaire distributed to community-based women, and participation was voluntary.

Respondents completed the questionnaire independently based on their personal perceptions and symptom experience. Menopausal status was established according to the STRAW+10 criteria based on menstrual history and self-reported reproductive status [3]. No dental examination, oral-health screening, or clinical dental procedures were performed as part of the study protocol.

Inclusion criteria were: age between 45 and 65 years, menopausal status according to STRAW+10 criteria, and ability to independently read and complete the questionnaire in Romanian. Exclusion criteria were: recent (<6 months) surgical or iatrogenic menopause; prior cervico-facial chemotherapy or radiotherapy; documented Sjögren’s syndrome or other autoimmune diseases associated with xerostomia; and acute psychiatric crisis at enrolment.

The distribution of menopausal stages (postmenopause, *n* = 202; 65.6%; perimenopause, *n* = 66; 21.4%; menopause, *n* = 40; 13.0%) was consistent with the expected age structure of community-based samples in this age range.

The sample size was fixed a priori by the requirements of the exploratory factor analysis. Three conventional criteria were applied: an absolute sample size of *N* ≥ 300, conventionally regarded as good for factor recovery; a subject-to-item ratio of at least 5–10, with 308 respondents and 44 items giving approximately seven respondents per item; and the requirement that, with communalities in the moderate range and well-overdetermined factors, samples of 200–300 are adequate for stable recovery. For internal consistency, *N* = 308 exceeds by a wide margin the sample of 100 required for a very good rating under COSMIN [31]. For the known-groups comparisons the smallest stratum (*n* = 40) is the limiting factor; power for stage-based contrasts is correspondingly modest, and non-significant results in that analysis should not be read as evidence of absence.

Three distinct groups contributed to the validation of the instrument. Content validity was established by a multidisciplinary expert panel of six clinicians (two gynecologists, two oral-medicine specialists, one clinical psychologist, one internist). Face validity and comprehensibility were assessed in a pilot sample of twelve women drawn from the target population. Psychometric validation was carried out in the main cohort of 308 community-dwelling women described above.

### 2.3. Instrument Development

The MMBQ-44 is a new instrument and the present study is its first administration; it was not, however, created de novo, nor was it adapted from any single existing questionnaire. It was assembled by deductive item generation, in which items were drawn from, adapted from, or modelled on validated instruments, and was then taken through three sequential development phases, each with a distinct purpose: expert review, to establish content validity; cognitive interviewing, to establish that items were understood as intended; and field administration, to generate the data required for psychometric evaluation. The contribution of the instrument is therefore integrative rather than novel at item level.

#### 2.3.1. Phase 1—Item Generation and Content Validity

The Multidimensional Menopausal Burden Questionnaire (MMBQ-44) was developed using a deductive item-generation approach grounded in the literature on menopausal symptomatology and estrogen deficiency. The full 44-item questionnaire, including item wording and response scales, is provided as Supplementary File S1.

Items were derived from, adapted from, or modelled on validated instruments, including the Menopause Rating Scale [33], the Xerostomia Inventory [22], the Oral Health Impact Profile short form [23], and psychological screening tools including the Patient Health Questionnaire-9 [25], the Generalized Anxiety Disorder-7 scale [26], and the Hospital Anxiety and Depression Scale [27].

A multidisciplinary expert panel (two gynecologists, two oral-medicine specialists, one clinical psychologist, one internist) reviewed each item for content relevance, scope, redundancy, and clinical interpretability. Item-level Content Validity Indices (I-CVI) and the Scale-level Content Validity Index averaged across the panel (S-CVI/Ave) were computed; only items reaching I-CVI ≥ 0.78 and contributing to S-CVI/Ave ≥ 0.90 were retained [34].

#### 2.3.2. Phase 2—Cognitive Interviewing and Face Validity

The pre-final 44-item version was administered to a pilot sample of 12 women drawn from the target population, who completed the questionnaire under a “think-aloud” cognitive-interviewing protocol [35].

Probes targeted comprehension, item recall, decision processes, and response mapping. Items flagged by ≥2 participants for ambiguity, double-barrelling, or culturally loaded phrasing were revised. Estimated administration time after pilot revision was 8–10 min.

#### 2.3.3. Phase 3—Field Administration and Covariates

Field testing, in the sense used here, denotes administration of the finalized 44-item instrument to the full validation cohort (*n* = 308) under the conditions of intended use—self-administered, community-based and without clinician assistance. It is distinct from the pilot phase in that no further item modification was performed after it. Sociodemographic and clinical covariates collected alongside the instrument were: age, height, weight, body mass index (BMI; computed and recoded to WHO categories) [36], educational attainment, smoking status, alcohol use and frequency, frequency of physical activity, time since last menstruation, menopausal stage, current hormone replacement therapy (HRT) and the specific preparation, polypharmacy, and use of medications with xerostomia-inducing potential (anticholinergics, antidepressants, antihypertensives).

### 2.4. Domain Composition and Scoring

The 44 items of the MMBQ were grouped a priori into four conceptual domains reflecting distinct dimensions of menopausal burden. The MRS domain comprised 18 items scored on a Likert scale from 0 to 4 (raw score range 0–72), capturing vasomotor, somatic, and urogenital symptoms. The XI domain included 8 items scored from 0 to 3 (raw range 0–24), assessing xerostomia and associated sensory alterations. The OHIP domain consisted of 9 items scored from 0 to 3 (raw range 0–27), reflecting oral-health-related functional impact. The PSY domain included 9 items scored from 0 to 4 (raw range 0–36), measuring psychological burden.

To ensure comparability across domains with different score ranges, all raw composite scores were linearly transformed to a standardized 0–100 scale using the formula (raw score/maximum possible score × 100). The total menopausal burden score was calculated as the unweighted sum of the four standardized domain scores. For inferential analyses, the total score was categorized into tertiles representing low, moderate, and severe burden, and used in known-groups comparisons and regression models.

### 2.5. Statistical Analysis

Continuous variables are reported as mean ± standard deviation (SD) or median (interquartile range, IQR), depending on distribution assessed using the Shapiro–Wilk test and the Kolmogorov–Smirnov test. Categorical variables are presented as absolute and relative frequencies.

Item-level psychometric performance was evaluated using descriptive statistics (mean, SD), distributional indices (skewness, kurtosis), the proportion of floor and ceiling effects, and missing data rates.

Internal consistency reliability was assessed using Cronbach’s α and McDonald’s ω [37], with values ≥ 0.70 considered acceptable and ≥0.80 good. Corrected item–total correlations (Spearman) were examined, with values ≥ 0.30 indicating adequate item discrimination. The effect of item removal on α was also evaluated.

Factorability of the full 44-item battery was assessed using the Kaiser–Meyer–Olkin (KMO) measure of sampling adequacy (threshold ≥ 0.60) and Bartlett’s test of sphericity (*p* < 0.05). Exploratory factor analysis (EFA) was conducted using principal axis factoring (PAF) with promax oblique rotation (κ = 4). The number of factors was determined using Horn’s parallel analysis [38], based on 1000 randomly generated datasets and the 95th-percentile eigenvalue criterion. Item retention and factor assignment were based on a primary loading threshold of ≥0.40, with cross-loadings ≥ 0.30 considered substantial and flagged for evaluation.

Convergent and discriminant validity were assessed using the average inter-item correlation within each domain (optimal range 0.15–0.50) and the heterotrait–monotrait (HTMT) ratio of correlations [39]. HTMT values < 0.85 (conservative) and <0.90 (lenient) were interpreted as evidence of discriminant validity.

Known-groups validity was evaluated using Kruskal–Wallis analysis of variance across menopausal stages, followed by Dunn–Bonferroni post hoc tests. Effect sizes were reported using epsilon-squared (ε^2^), interpreted as small (≥0.01), moderate (≥0.06), and large (≥0.14). Because the three stage groups differ substantially in size, the known-groups comparison was repeated as a sensitivity analysis in 1000 random subsamples matched at *n* = 40 per stage. Floor and ceiling effects at the composite (domain) level were considered present if ≥15% of respondents achieved the minimum or maximum possible scores, respectively.

All statistical tests were two-sided, with a significance threshold set at α = 0.05. All analyses were performed in R (version 4.3.2) using the psych, GPArotation, paran and lavaan packages.

## 3. Results

### 3.1. Sample Characteristics

The 308 women had a mean age of 53.4 ± 5.5 years and a mean body mass index (BMI) of 27.3 ± 5.3 kg/m^2^, with 37% classified as overweight according to World Health Organization criteria. Stage-specific characteristics are summarized in Table 1. Hormone replacement therapy (HRT) uptake was low (6.2%), and 12.7% of participants reported the use of at least one medication with documented xerostomia-inducing potential—both relevant covariates for the interpretation of oral-domain findings.

### 3.2. Item-Level Psychometric Performance

Item-level descriptive statistics indicated non-normal distributions, with positive skewness particularly evident in the XI and OHIP domains, where several items exhibited modal responses at the lowest category. Missing data were negligible across all 44 items (Supplementary Table S1).

At the domain level (Table 2), all four domains demonstrated strong internal consistency. Cronbach’s α and McDonald’s ω exceeded 0.80 across domains, with the MRS and PSY domains showing values above 0.90, indicative of excellent reliability without exceeding the >0.95 threshold suggestive of redundancy. Average inter-item correlations were within acceptable ranges, supporting internal coherence.

Corrected item–total Spearman correlations (Table 3) exceeded the conventional 0.30 threshold for all items across all domains, indicating adequate item discrimination. The lowest observed value (ρ = 0.379) was recorded for the OHIP item *“Mouth ulcers or sores.”*.

Item-deletion analyses (Table 3) indicated that removal of any item did not meaningfully improve internal consistency. Only one item—“*I have difficulty falling asleep or I wake up often for no reason*” (PSY domain)—yielded a positive change in Cronbach’s α; however, this increase was trivial (Δα = +0.003) and well below the threshold for meaningful improvement (Δα > 0.01). Given its conceptual overlap with the MRS sleep-related item, this finding likely reflects content redundancy rather than psychometric weakness, and revision rather than deletion is recommended.

### 3.3. Factorability and Exploratory Factor Analysis

The full 44-item battery demonstrated excellent factorability, with a Kaiser–Meyer–Olkin (KMO) value of 0.931 and a significant Bartlett’s test of sphericity (χ^2^(946) = 9006.2, *p* < 0.001). Domain-specific KMO values were also high (MRS = 0.912, XI = 0.824, OHIP = 0.832, PSY = 0.936).

**Figure 1:**
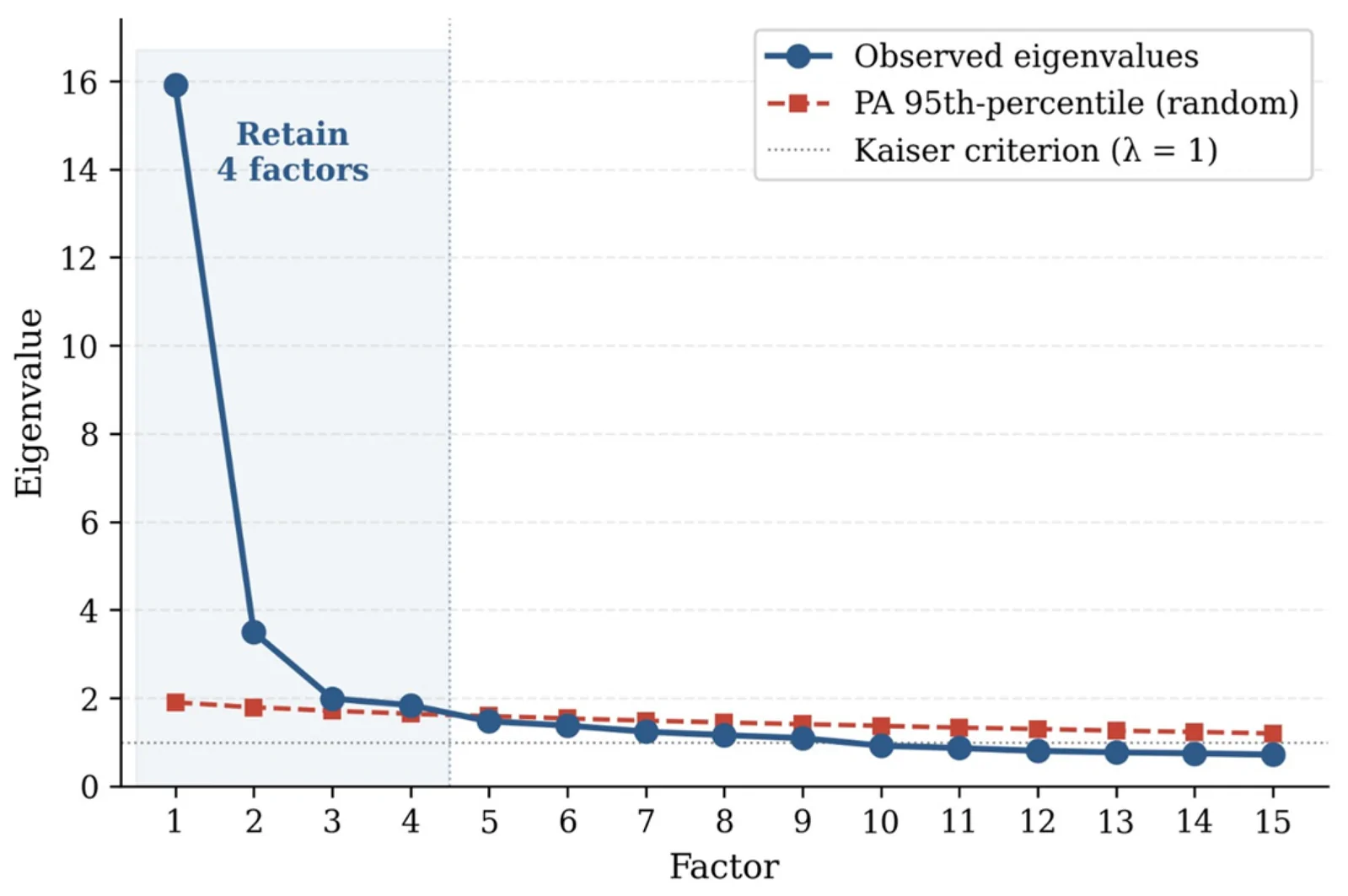
Scree plot of observed eigenvalues and Horn’s parallel analysis for factor retention. Observed eigenvalues (solid line) are compared with eigenvalues derived from randomly generated datasets (dashed line; 95th percentile). The dotted horizontal line indicates the Kaiser criterion (eigenvalue = 1). Factors are retained where observed eigenvalues exceed the parallel analysis threshold.

Horn’s parallel analysis supported the retention of four factors, consistent with the a priori domain structure. The factor retention decision is illustrated in Figure 1, where the first four observed eigenvalues exceed the 95th-percentile random eigenvalues.

The four-factor solution accounted for 48.61% of the total variance after promax rotation. Forty of the 44 items exhibited primary loadings ≥ 0.40, while seven items showed cross-loadings ≥ 0.30. The four items whose primary loading fell below 0.40 were decreased interest in sexual activity (MRS; λ = 0.321, assigned to F4), vaginal dryness, itching or burning (MRS; λ = 0.398, assigned to F1, with a secondary loading of 0.378 on F4), nocturnal thirst (XI; λ = 0.386, assigned to F2), and food tastes different than it used to be (XI; λ = 0.349, assigned to F3). Each was assigned to the factor on which it loaded most strongly. All four were retained rather than deleted, because each retained an adequate corrected item–total correlation within its a priori domain (all ≥ 0.30), deletion of none of them improved Cronbach’s α by a meaningful margin, and each represents a clinically non-redundant facet of menopausal burden; they are, however, the primary candidates for revision or replacement before confirmatory validation.

**Figure 2:**
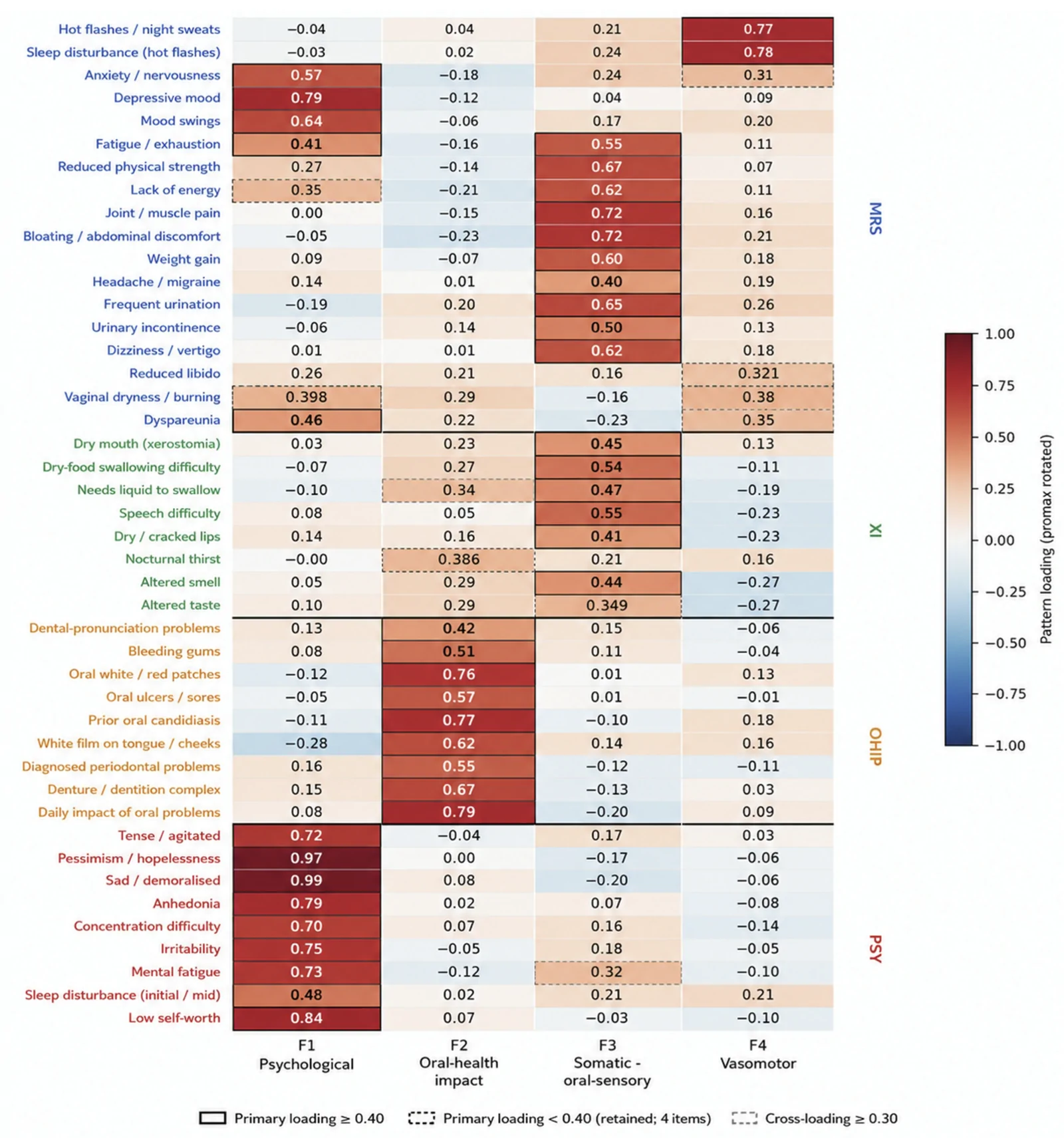
Rotated factor-loading matrix of the MMBQ-44 (principal axis factoring, promax rotation; *N* = 308). Rows are the 44 items, grouped by a priori domain and separated by horizontal rules; row labels are coloured by domain (MRS, blue; XI, green; OHIP, amber; PSY, red). Columns are the four extracted factors, labelled by the content they actually recovered. Cell colour encodes the pattern loading on a diverging scale centred on zero, so that positive and negative loadings are immediately distinguishable, and the numerical loading is printed in every cell. A solid border marks a primary loading at or above the 0.40 retention threshold; a dotted border marks the four items whose primary loading falls below it (shown to three decimals); a dashed grey border marks a cross-loading of 0.30 or more on a factor other than the item’s own. Read row-wise, the figure shows where each item loads; read column-wise, it shows how far each empirical factor corresponds to the a priori domain of the items composing it. MRS, Menopause Rating Scale domain; XI, Xerostomia Inventory domain; OHIP, Oral Health Impact Profile domain; PSY, psychological domain.

The rotated factor-loading structure is presented in Figure 2, which maps empirical loadings against the a priori domain classification.

Empirical factor composition (Table 4) showed partial alignment with the predefined domains. The first factor (F1) corresponded primarily to psychological items but also incorporated five MRS items related to mood and sexual symptoms. The second factor (F2) recovered the OHIP block in full, together with a single Xerostomia Inventory item (nocturnal thirst, λ = 0.386) whose primary loading fell below the retention threshold. The third factor (F3) combined MRS somatic and XI items, indicating a fusion of systemic and oral-sensory burden. The fourth factor (F4) was a vasomotor factor, comprising hot flashes or night sweats (λ = 0.765), difficulty sleeping due to hot flashes (λ = 0.777) and decreased interest in sexual activity (λ = 0.321); the urinary items loaded on F3, and vaginal dryness and dyspareunia on F1.

These findings suggest that while the overall dimensionality of the instrument is preserved, the boundaries between somatic, oral, and psychological domains are partially overlapping. The full rotated factor-loading matrix is provided in Supplementary Table S2.

Items are grouped by extracted factors and compared with their original (a priori) domain classification. Factor extraction was performed using principal axis factoring with promax oblique rotation. Alignment indicates the degree of correspondence between empirical factor structure and theoretical domain assignment. MRS, Menopause Rating Scale domain; XI, Xerostomia Inventory domain; OHIP, Oral Health Impact Profile domain; PSY, psychological domain.

### 3.4. Convergent and Discriminant Validity

Domain-level associations are summarized in Table 5, which presents both Spearman intercorrelations (ρ) and heterotrait–monotrait (HTMT) ratios in a single matrix. All domain composites were positively intercorrelated, with the strongest association observed between MRS and PSY (ρ = 0.79) and the weakest between MRS and OHIP (ρ = 0.45).

Lower triangle presents Spearman correlation coefficients (ρ), indicating the strength of association between domain composite scores. Upper triangle presents heterotrait–monotrait (HTMT) ratios, used to assess discriminant validity between constructs. Values < 0.85 (conservative threshold) and <0.90 (lenient threshold) indicate acceptable discriminant validity. MRS, Menopause Rating Scale domain; XI, Xerostomia Inventory domain; OHIP, Oral Health Impact Profile domain; PSY, psychological domain.

**Figure 3:**
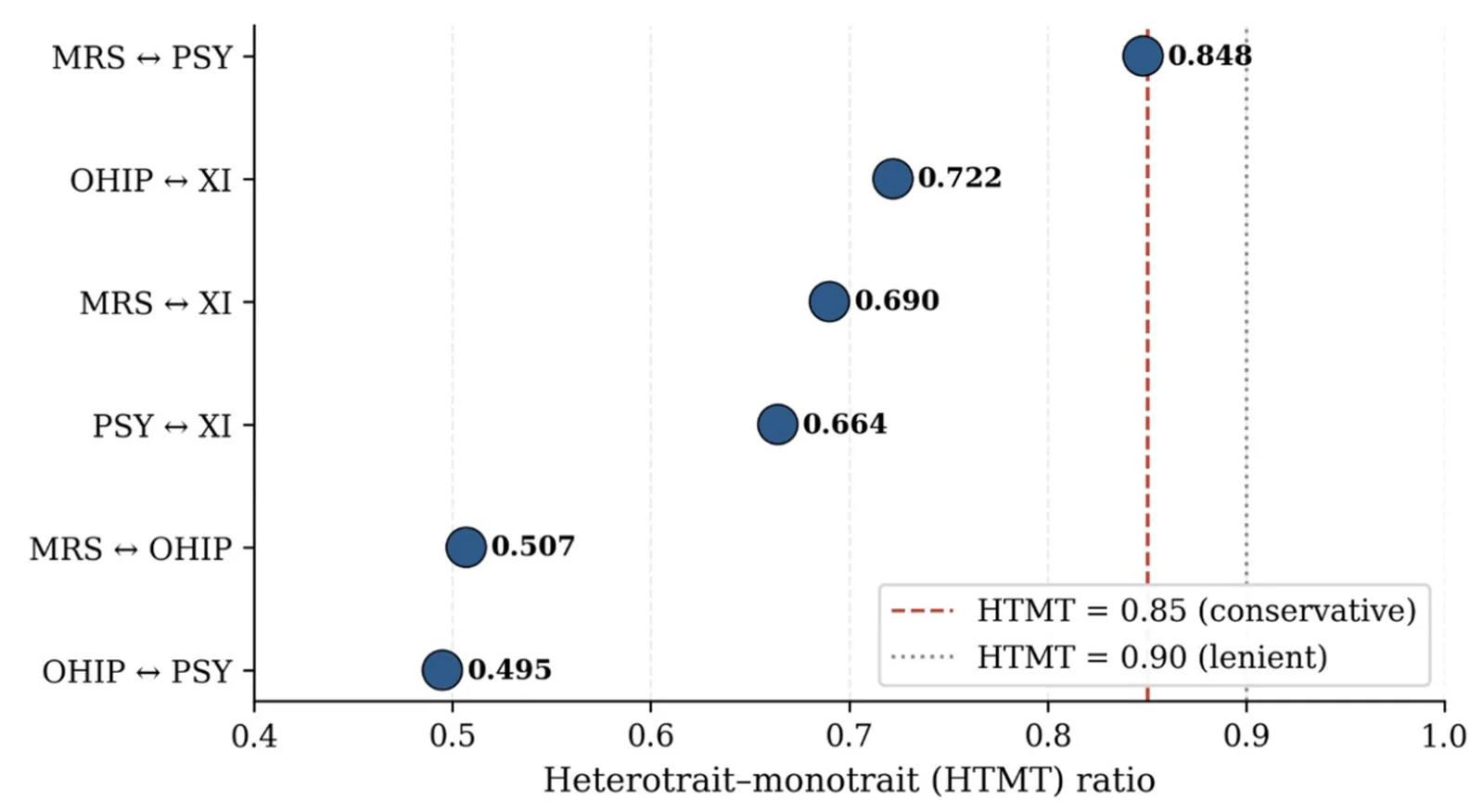
Heterotrait–monotrait (HTMT) ratios between MMBQ-44 domains. HTMT ratios are displayed for all domain pairs. The dashed red line represents the conservative threshold (0.85), and the dotted grey line represents the lenient threshold (0.90). Values below these thresholds support discriminant validity. MRS, Menopause Rating Scale domain; XI, Xerostomia Inventory domain; OHIP, Oral Health Impact Profile domain; PSY, psychological domain.

HTMT ratios supported discriminant validity for most domain pairs. Five of the six HTMT values were below the conservative threshold of 0.85, while the MRS–PSY pair (HTMT = 0.848) approached this threshold but remained below the lenient criterion of 0.90. This borderline result mirrors the EFA findings, where partial overlap between psychological and somatic dimensions was observed. To facilitate interpretation, HTMT ratios are visualized in Figure 3.

These findings indicate that, although the four domains are conceptually distinct, moderate inter-domain associations exist, particularly between somatic and psychological burden.

At the item level, the mucosal-convergence hypothesis was supported, with xerostomia scores correlating with vaginal dryness (ρ = 0.384, *p* < 0.001) and dyspareunia (ρ = 0.317, *p* < 0.001). The affective-amplification hypothesis was also confirmed, with a strong association between xerostomia (XI) scores and psychological burden (ρ = 0.610, *p* < 0.001).

### 3.5. Known-Groups Validity

Differences across menopausal stages were assessed using Kruskal–Wallis tests (Table 6). Only the OHIP composite demonstrated statistically significant variation (H = 6.85, df = 2, *p* = 0.033, ε^2^ = 0.016), with post hoc comparisons indicating higher scores in postmenopausal compared with perimenopausal women.

No significant differences were observed for MRS, XI, or PSY composites. This pattern suggests that menopausal burden is already established in the perimenopausal stage and does not increase linearly across stages.

In the sensitivity analysis using 1000 subsamples matched at *n* = 40 per menopausal stage, OHIP remained the domain with the largest stage-related difference, but the comparison reached statistical significance in only 12.0% of subsamples (MRS 5.1%, XI 0.9%, PSY 0.0%). Matching the groups would therefore not change the substantive finding, but it would remove the statistical power required to detect it; this is the principal reason the full unbalanced sample was retained for analysis.

Values are presented as median (interquartile range). Group differences were tested using the Kruskal–Wallis test. ε^2^ (epsilon-squared) is reported as effect size. Post hoc comparisons were performed using Dunn–Bonferroni correction. MRS, Menopause Rating Scale domain; XI, Xerostomia Inventory domain; OHIP, Oral Health Impact Profile domain; PSY, psychological domain. Epsilon-squared values that were negative through sampling variation are bounded below by zero and are reported as <0.001.

### 3.6. Floor and Ceiling Effects

Composite-level floor and ceiling effects are summarized in Table 7. No ceiling effects were observed for any domain.

Floor effects exceeded the 15% threshold for the OHIP (37.0%) and XI (23.4%) domains, indicating a substantial proportion of participants with minimal oral or sensory symptom burden. This pattern is consistent with the use of a community-based sample and suggests reduced sensitivity of these domains at the lower end of the symptom spectrum.

Floor and ceiling effects represent the percentage of participants achieving the minimum or maximum possible scores, respectively. Values ≥ 15% are considered indicative of potential limitations in measurement sensitivity. MRS, Menopause Rating Scale domain; XI, Xerostomia Inventory domain; OHIP, Oral Health Impact Profile domain; PSY, psychological domain.

## 4. Discussion

This study describes the development and initial psychometric validation of the Multidimensional Menopausal Burden Questionnaire (MMBQ-44), an instrument designed to integrate systemic, oral-sensory (xerostomia), oral-health, and psychological dimensions of menopausal burden within a unified framework. In a community-based cohort of 308 Romanian women, the instrument showed strong internal consistency across all domains, excellent factorability, and a four-factor structure broadly consistent with the conceptual model, including a distinct vasomotor factor. Item performance was uniformly adequate, with no relevant missing data, satisfactory item–total correlations for all 44 items, and minimal impact of item deletion. At the same time, the exploratory factor analysis, the strong correlation between the systemic and psychological domains, and the borderline HTMT value revealed meaningful cross-domain associations, indicating that menopausal symptom domains are partially overlapping rather than fully independent, and that the MMBQ-44 reflects a partially shared latent structure of menopausal burden.

Several findings converge with previous research. The observed systemic–psychological overlap is consistent with contemporary menopause care models that favour holistic, biopsychosocial frameworks over symptom-specific evaluation [40], and with current clinical guidance recommending that menopausal symptoms be assessed and managed on the basis of overall burden rather than isolated complaints [29,41]. Mechanistically, fluctuations in estrogen levels influence serotonergic and hypothalamic pathways, linking vasomotor symptoms, sleep disturbance, and affective symptoms within a shared physiological axis [5], and neuroendocrine modulation of mood and behaviour during the menopausal transition is well documented [6]. Recent clinical studies confirm that these symptoms frequently co-occur and are strongly interrelated across the menopausal transition [42], and network analyses similarly demonstrate cross-domain symptom clustering [43]; from a psychometric standpoint, this degree of overlap is expected when constructs share underlying variance [44,45]. The partial convergence of oral-sensory items with the systemic and psychological domains is likewise supported by prior evidence: xerostomia is multifactorial, influenced by hormonal changes, medications, systemic disease, and psychological factors [10]; estrogen decline directly affects salivary gland function and mucosal homeostasis [8]; psychological stress alters salivary function through autonomic imbalance and hypothalamic–pituitary–adrenal axis activation [46]; and clinical studies consistently report associations between xerostomia, anxiety, and depression [47], reinforcing the importance of assessing psychological burden in patients presenting with oral complaints [48]. The association between xerostomia and genital symptoms further supports the concept of mucosal convergence across estrogen-sensitive tissues, in line with the recognition of the genitourinary syndrome of menopause as a multisite condition [49], suggesting that oral-sensory symptoms could act as accessible markers facilitating earlier recognition of genitourinary or systemic involvement [50].

Other findings warrant a more nuanced comparison with the literature. Floor effects were present in the oral-health and xerostomia domains, limiting discrimination among women with minimal oral symptoms; this pattern is consistent with epidemiological evidence that xerostomia in the general population is usually mild or intermittent outside clinical settings, and with the expectation that multidetermined symptoms load onto broader factors in low-symptom community samples [11,45]. In this sample, the high prevalence of polypharmacy (41.2%) and of xerostomia-inducing medications (12.7%) likely added variability in oral-sensory symptoms beyond menopausal stage alone [10]. By contrast, the absence of ceiling effects across all domains indicates preserved sensitivity across the upper range of symptom intensity, supporting utility in populations with more pronounced burden [51]. Known-groups comparisons showed only limited differences across menopausal stages, suggesting that symptom burden is not strictly stage-dependent but shaped by biological, behavioural, and contextual factors, with the increased oral-health impact in postmenopausal women possibly reflecting cumulative hormonal deficiency or delayed clinical manifestation. Importantly, integrating items from established instruments did not compromise reliability [52], and the slight increase in Cronbach’s α after deletion of the psychological sleep item is more plausibly explained by conceptual overlap with the MRS sleep item than by deficient item performance. Taken together, these results support a reconceptualization of menopausal burden as a multidimensional construct with partially overlapping domains [28], favouring parallel, multidomain assessment over sequential symptom evaluation in clinical workflows [53] and supporting multidisciplinary, integrated evaluation strategies [29].

This study has both strengths and limitations. Strengths include the community-based design, the absence of relevant missing data, the consistent performance across complementary psychometric indicators, and the successful integration of validated instruments within a single multidomain framework. Limitations include the cross-sectional design, which precludes conclusions on causality, symptom trajectories, or responsiveness to change; the absence of biochemical markers, which prevents direct correlation between symptom clusters and hormonal status; the relatively small subsamples for certain menopausal stages, increasing the risk of type II error in stage-based comparisons; and the single-centre, community-based sample, which may limit generalizability to more symptomatic clinical populations. From a psychometric perspective, the lack of confirmatory factor analysis in an independent sample and of test–retest reliability assessment limits conclusions on structural stability and temporal consistency, the observed floor effects suggest reduced sensitivity in low-symptom populations, reliance on self-reported data introduces potential reporting bias, and four items that did not reach the primary-loading threshold of 0.40 were retained at this initial stage but remain the primary candidates for revision or replacement before confirmatory validation.

In conclusion, this initial validation provides preliminary evidence that systemic, oral, and psychological aspects of menopausal burden can be reliably assessed within a single coherent framework, although the MMBQ-44 should not yet be used for diagnostic classification until longitudinal stability and clinically meaningful thresholds are established. Future research should focus on confirmatory and longitudinal validation in larger, more diverse, and more symptomatic populations, integration of objective biomarkers, and item-response theory modelling to identify items with optimal discriminatory capacity across severity levels. Of particular interest is the potential predictive value of oral-sensory symptoms for broader menopausal syndromes, including genitourinary and psychological conditions; if confirmed, this would position the oral domain as a clinically useful entry point for multidimensional menopause assessment.

## 5. Conclusions

The MMBQ-44 demonstrated good-to-excellent internal consistency, adequate factorability, and a four-factor structure broadly consistent with the a priori model in a sample of 308 women aged 45–65 years. Some divergence between empirical and theoretical domain composition was observed, particularly in the partial overlap between somatic and psychological items and the integration of oral-sensory items within the somatic factor.

Discriminant validity was supported for most domain pairs, although the association between systemic and psychological domains approached the conservative threshold. These findings suggest that certain symptom domains may not be fully independent and may reflect partially shared underlying constructs. The oral-sensory domain showed moderate associations with both systemic and psychological domains, indicating potential overlap in symptom reporting across these areas.

The MMBQ-44 may provide a structured approach for assessing multiple dimensions of menopausal symptoms within a single instrument. Further studies are required to confirm the factor structure, evaluate temporal stability, and examine performance in different populations and clinical settings, including the incorporation of objective clinical and biological measures.

## Figures and Tables

**Table 1:** Sociodemographic and clinical characteristics of the study sample by menopausal stage (*N* = 308).

Variable	Total (*N* = 308)	Perimenopause (*n* = 66)	Menopause (*n* = 40)	Postmenopause (*n* = 202)	*p*-Value
Age (years), median (IQR)	52 (49–58)	49 (47–50)	48 (46–51.3)	56.5 (52–59)	<0.001
Height (cm), median (IQR)	164 (160–169)	166.5 (162–170)	164 (160–169.3)	163 (160–168)	0.060
Weight (kg), median (IQR)	72 (64–82)	67 (63.3–80.5)	71 (62–78.5)	75 (65–83)	0.030
BMI (kg/m^2^), median (IQR)	26.8 (23.5–30.1)	24.8 (22.8–28.7)	26.3 (21.9–29.8)	27.5 (24.2–30.8)	0.004
Overweight (BMI 25.0–29.9), *n* (%)	114 (37.0)	20 (30.3)	14 (35.0)	80 (39.6)	0.382
Obesity (BMI ≥ 30), *n* (%)	85 (27.6)	12 (18.2)	9 (22.5)	64 (31.7)	0.077
Years since last menstruation, median (IQR)	4 (1–8)	0.00 (0–0)	1 (1–1)	6 (4–10)	<0.001
Tertiary education, *n* (%)	213 (69.2)	57 (86.4)	27 (67.5)	129 (63.9)	0.003
Current smoker, *n* (%)	74 (24.0)	16 (24.2)	14 (35.0)	44 (21.8)	0.202
Any alcohol consumption, *n* (%)	72 (23.4)	15 (22.7)	7 (17.5)	50 (24.8)	0.606
Current HRT, *n* (%)	19 (6.2)	6 (9.1)	3 (7.5)	10 (5.0)	0.446
Daily polypharmacy (≥2 drugs), *n* (%)	127 (41.2)	20 (30.3)	9 (22.5)	98 (48.5)	0.001
Xerostomia-inducing medications, *n* (%)	39 (12.7)	4 (6.1)	2 (5.0)	33 (16.3)	0.027

*p*-values for between-stage comparisons: Kruskal–Wallis test for continuous variables (age, height, weight, BMI, years since last menstruation) and Pearson χ^2^ test for categorical variables (with Fisher’s exact correction when expected counts < 5). Values are presented as median, IQR (interquartile range formula) for continuous variables that are not normally distributed and as number (percentage) for categorical variables. Menopausal stages were defined according to the STRAW+10 criteria. BMI, body mass index; HRT, hormone replacement therapy. Polypharmacy was defined as the concurrent use of two or more medications. Xerostomia-inducing medications include drug classes known to reduce salivary flow (e.g., anticholinergics, antidepressants, antihypertensives). The Shapiro–Wilk test rejected normality for every continuous variable in this table (age *p* = 6.2 × 10^−8^; height *p* = 0.009; weight *p* = 5.6 × 10^−6^; BMI *p* = 9.6 × 10^−8^; years since last menstruation *p* = 1.0 × 10^−13^), which is why the median and interquartile range are reported here rather than the mean and standard deviation. The corresponding means and standard deviations are given in Supplementary Table S3.

**Table 2:** Internal consistency and item–total correlations for the four a priori domains of the MMBQ-44 (*N* = 308).

Domain	k Items	Cronbach’s α	McDonald’s ω	Avg. Inter-Item r	Item–Total ρ Range
MRS—somato-vasomotor-urogenital	18	0.927	0.937	0.407	0.434–0.776
XI—xerostomia/ sensory	8	0.813	0.864	0.380	0.463–0.595
OHIP—oral-health impact	9	0.815	0.868	0.340	0.379–0.586
PSY—psychological	9	0.941	0.953	0.632	0.665–0.830

Cronbach’s α and McDonald’s ω are reported as measures of internal consistency reliability. Avg. inter-item r: average inter-item correlation within each domain. Item–total ρ range: range of corrected item–total Spearman correlations for items within each domain. MRS, Menopause Rating Scale domain; XI, Xerostomia Inventory domain; OHIP, Oral Health Impact Profile domain; PSY, psychological domain.

**Table 3:** Corrected item–total correlations (Spearman ρ between item and rest-of-domain total) and Cronbach’s α-if-deleted, by a priori domain.

Item	Item–Total ρ	α if Deleted	Δα
MRS domain (k = 18; full-domain α = 0.927)			
Hot flashes or night sweats	0.551	0.925	−0.0023
Difficulty sleeping due to hot flashes	0.563	0.924	−0.0028
Anxiety or nervousness	0.750	0.920	−0.0069
Feeling depressed	0.657	0.922	−0.0047
Mood swings	0.710	0.920	−0.0065
Fatigue or exhaustion	0.776	0.919	−0.0080
Decrease in physical strength	0.747	0.920	−0.0068
Lack of energy	0.765	0.920	−0.0073
Joint or muscle pain	0.605	0.923	−0.0036
Bloating/abdominal discomfort/gallbladder attacks	0.523	0.925	−0.0020
Weight gain	0.596	0.923	−0.0039
Headache (migraine, headache)	0.514	0.925	−0.0023
Frequent urination	0.611	0.923	−0.0035
Urinary incontinence (when coughing or without coughing)	0.451	0.926	−0.0010
Dizziness, vertigo, or balance problems	0.582	0.923	−0.0036
Decreased interest in sexual activity	0.607	0.923	−0.0039
Vaginal dryness, itching, burning	0.501	0.925	−0.0021
Discomfort/pain during intercourse	0.434	0.926	−0.0009
XI domain (k = 8; full-domain α = 0.813)			
Feeling of “dry mouth”	0.595	0.785	−0.0274
Difficulty swallowing dry food	0.561	0.785	−0.0279
The need to sip liquids in order to swallow	0.531	0.786	−0.0268
Speech difficulties	0.504	0.796	−0.0171
Dry, cracked lips	0.463	0.802	−0.0103
Nocturnal thirst	0.514	0.804	−0.0085
Changes in smell	0.554	0.784	−0.0287
Food tastes different than it used to be	0.520	0.789	−0.0235
OHIP domain (k = 9; full-domain α = 0.815)			
Dental problems causing difficulty pronouncing letters/words	0.458	0.803	−0.0116
Gums are sore when brushing/eating	0.586	0.797	−0.0182
White or red spots in the oral cavity	0.477	0.791	−0.0240
Mouth ulcers or sores	0.379	0.804	−0.0110
History of oral candidiasis	0.413	0.799	−0.0162
White coating on the tongue or cheeks	0.422	0.801	−0.0141
Diagnosed periodontal disease	0.510	0.803	−0.0117
Complexes related to teeth or dentures	0.573	0.783	−0.0313
Difficulty in daily activities due to oral problems	0.509	0.785	−0.0298
PSY domain (k = 9; full-domain α = 0.941)			
I feel tense, nervous, or agitated	0.781	0.934	−0.0071
I have pessimistic thoughts or feel hopeless	0.749	0.933	−0.0085
I feel sad or demoralized	0.791	0.932	−0.0096
I lose interest in activities that I used to enjoy	0.740	0.935	−0.0070
I have difficulty concentrating	0.740	0.935	−0.0064
I feel irritable or “easily provoked”	0.803	0.933	−0.0081
I feel mentally tired, without energy	0.830	0.932	−0.0100
I have difficulty falling asleep or I wake up often for no reason	0.665	0.944	0.0025
I feel worthless or have lost self-confidence	0.777	0.934	−0.0075

Item–total ρ, corrected Spearman correlation between each item and the sum of the remaining items within the same domain (i.e., item excluded from the total score); α if deleted, Cronbach’s α of the domain after removal of the respective item; Δα, change in Cronbach’s α relative to the full-domain value (positive values indicate an increase in α upon item deletion); k, number of items in the domain.

**Table 4:** Empirical factor structure of the MMBQ-44 compared with a priori domain assignment (principal axis factoring with promax rotation; *N* = 308).

Empirical Factor	Items (*n*)	A Priori Source— Predominant	Alignment
F1 (Psychological)	14	9 PSY + 5 MRS items	Mostly PSY, plus MRS mood/sexual items
F2 (OHIP)	10	9 OHIP + 1 XI items	Complete OHIP block plus one XI item (nocturnal thirst)
F3 (Somatic–oral-sensory)	17	10 MRS + 7 XI items	XI items fuse with MRS somatic block
F4 (Vasomotor)	3	3 MRS items	Hot flashes, hot-flash-related sleep disturbance, decreased sexual interest

**Table 5:** Domain intercorrelations (Spearman ρ) and heterotrait–monotrait (HTMT) ratios of the MMBQ-44 domains (*N* = 308).

Domain	MRS	XI	OHIP	PSY
MRS	1.00	0.690	0.507	0.848
XI	0.65	1.00	0.722	0.664
OHIP	0.45	0.56	1.00	0.495
PSY	0.79	0.61	0.48	1.00

**Table 6:** Known-groups validity: comparison of domain scores (0–100 scale) across menopausal stages (*N* = 308).

Domain	Total	Perimenopause (*n* = 66)	Menopause (*n* = 40)	Postmenopause (*n* = 202)	Kruskal–Wallis H Statistic	*p*-Value	ε^2^
MRS	27.78 (16.67–48.61)	22.92 (11.46–46.88)	31.25 (21.88–52.78)	27.78 (18.06–48.61)	3.48	0.176	0.005
XI	8.33 (4.17–25.00)	8.33 (0.00–25.00)	12.50 (3.13–25.00)	8.33 (4.17–25.00)	1.37	0.503	<0.001
OHIP	5.56 (0.00–14.81)	1.85 (0.00–10.19)	3.70 (0.00–14.81)	7.41 (0.00–14.81)	6.85	0.033	0.016
PSY	25.00 (11.11–42.36)	22.22 (11.11–49.31)	27.78 (8.33–53.47)	23.61 (11.11–38.89)	0.24	0.887	<0.001

**Table 7:** Floor and ceiling effects for MMBQ-44 domain scores (*N* = 308).

Domain	Range	Mean ± SD	% at Floor	% at Ceiling
MRS	0–72	23.53 ± 14.80	2.27%	0.00%
XI	0–24	3.60 ± 3.67	23.38%	0.00%
OHIP	0–27	2.71 ± 3.48	37.01%	0.00%
PSY	0–36	10.51 ± 8.57	7.79%	0.00%

## Data Availability

The data presented in this study are available on request from the corresponding author. The data is not publicly available due to privacy and ethical restrictions.

## Supplementary Materials

The following supporting information can be downloaded at https://www.mdpi.com/article/10.3390/diagnostics16162574/s1, File S1: Patient questionnaire; Table S1: Item-level descriptive statistics for all 44 items of the MMBQ-44 (*N* = 308).; Table S2: Pattern matrix loadings for all 44 items on the four extracted factors; Table S3: Mean and standard deviation of the continuous characteristics reported in Table 1, by menopausal stage(*N* = 308); Table S4: Domain coverage of menopause-specific, oral-health and psychological instruments in current use, compared with the MMBQ-44.

## Abbreviations

The following abbreviations are used in this manuscript:

BMI	Body Mass Index
EFA	Exploratory Factor Analysis
GSM	Genitourinary Syndrome of Menopause
HADS	Hospital Anxiety and Depression Scale
HRT	Hormone Replacement Therapy
HTMT	Heterotrait–Monotrait Ratio
I-CVI	Item-level Content Validity Index
IRT	Item Response Theory
KMO	Kaiser–Meyer–Olkin Measure of Sampling Adequacy
MMBQ-44	Multidimensional Menopausal Burden Questionnaire (44 items)
MRS	Menopause Rating Scale
OHIP	Oral Health Impact Profile
PAF	Principal Axis Factoring
PHQ-9	Patient Health Questionnaire-9
PSY	Psychological Domain
SD	Standard Deviation
S-CVI/Ave	Scale-level Content Validity Index (Average)
STRAW+10	Stages of Reproductive Aging Workshop +10
XI	Xerostomia Inventory
