# Supplementary material for: Development and Initial Psychometric Validation of the Multidimensional Menopausal Burden Questionnaire (MMBQ-44) in a Sample of Romanian Women
Source: Diagnostics (Basel). 2026 Aug 14;16(16):2574. doi: 10.3390/diagnostics16162574 (PMC13512511; doi:10.3390/diagnostics16162574)
Supplement: Supplementary file 1 [file diagnostics-16-02574-s001.zip › File S1. PATIENT QUESTIONNAIRE.pdf]

**Supplementary File S1**
**PATIENT QUESTIONNAIRE**

**Medical research study – Clinical-biochemical correlations and psychological impact in perimenopause, menopause and postmenopause**
This questionnaire is confidential and has a strictly scientific purpose. Please answer the questions below honestly.

---

**1. General dates**

- Age: ___ years
- Height: ____ cm
- Weight: _____ kg
- Level of studies: ☐ High School ☐ Post-secondary ☐ Faculty ☐ Master ☐ Doctorate
- Do you smoke? ☐ Yes ☐ No
- Do you drink alcohol? ☐ Yes ☐ No (If yes, how often?)
  ☐ 1–2 times / week
  ☐ 3–5 times / week
  ☐ Daily
- How often do you exercise?
  ☐ Not at all
  ☐ 1–2 times / week
  ☐ 3–5 times / week
  ☐ Daily

**2. Medical history and hormonal status**

- How long has it been since your last period (years)? __________
- Are you currently using hormone replacement therapy? ☐ Yes ☐ No
  - If yes, please mention them: ______________________________
- Are you currently taking medication? ☐ Yes ☐ No
  - If yes, please mention them: ______________________________
- How many medications do you take daily and in what form of administration?
  ☐ I don't take medication for chronic
  ☐ conditions I only take medication for acute situations (viruses, bacterial infections, etc.)
  ☐ 1-2 tablets
  ☐ 3 or more tablets
  ☐ Injectable treatment (intramuscular/intravenous)
- Do you take medications that can cause a "dry mouth" sensation?
  - (ex: antihipertensive, antidepresive, etc.) ☐ Da ☐ Nu

**3. General symptoms (score: 0–4)**
**(0 = Not applicable, 1 = Slightly disturbing, 2 = Moderate, 3 = Very disturbing, 4 = Extremely disturbing)**

| **Symptom** | **0** | **1** | **2** | **3** | **4** |
|---|---|---|---|---|---|
| Hot flashes or night sweats | ☐ | ☐ | ☐ | ☐ | ☐ |
| Difficulty sleeping due to hot flashes | ☐ | ☐ | ☐ | ☐ | ☐ |
| Anxiety or nervousness | ☐ | ☐ | ☐ | ☐ | ☐ |
| Depressive states | ☐ | ☐ | ☐ | ☐ | ☐ |

| Symptom | 0 | 1 | 2 | 3 | 4 |
|---|---|---|---|---|---|
| Sudden mood swings | ☐ | ☐ | ☐ | ☐ | ☐ |
| Fatigue or exhaustion | ☐ | ☐ | ☐ | ☐ | ☐ |
| Decreased physical strength | ☐ | ☐ | ☐ | ☐ | ☐ |
| Lack of energy | ☐ | ☐ | ☐ | ☐ | ☐ |
| Joint or muscle pain | ☐ | ☐ | ☐ | ☐ | ☐ |
| Bloating / abdominal discomfort / gallbladder attacks (gallbladder) | ☐ | ☐ | ☐ | ☐ | ☐ |
| Weight gain | ☐ | ☐ | ☐ | ☐ | ☐ |
| Headache (migraine, headache) | ☐ | ☐ | ☐ | ☐ | ☐ |
| Frequent urination | ☐ | ☐ | ☐ | ☐ | ☐ |
| Urinary incontinence (with or without coughing) | ☐ | ☐ | ☐ | ☐ | ☐ |
| Dizziness/vertigo/balance disorders | ☐ | ☐ | ☐ | ☐ | ☐ |
| Decreased interest in sexual activity | ☐ | ☐ | ☐ | ☐ | ☐ |
| Vaginal dryness, itching, burning | ☐ | ☐ | ☐ | ☐ | ☐ |
| Discomfort/pain during intercourse | ☐ | ☐ | ☐ | ☐ | ☐ |

**4.Shipping: Oral symptoms and oral cavity**
**(0 = never, 1 = occasionally, 2 = frequently, 3 = always)**

| Symptom | 0 | 1 | 2 | 3 |
|---|---|---|---|---|
| The feeling of "dry mouth" | ☐ | ☐ | ☐ | ☐ |
| Difficulty swallowing dry food | ☐ | ☐ | ☐ | ☐ |
| The need to sip liquids to be able to swallow | ☐ | ☐ | ☐ | ☐ |
| Speech difficulties | ☐ | ☐ | ☐ | ☐ |
| Dry, cracked lips | ☐ | ☐ | ☐ | ☐ |
| Feeling of nocturnal thirst | ☐ | ☐ | ☐ | ☐ |
| Changes in smell | | | | |
| o Decrease | ☐ | ☐ | ☐ | ☐ |
| o partial disappearance | ☐ | ☐ | ☐ | ☐ |
| o total extinction | ☐ | ☐ | ☐ | ☐ |
| Food tastes different than it did before | ☐ | ☐ | ☐ | ☐ |
| Dental problems with difficulty pronouncing letters/words | ☐ | ☐ | ☐ | ☐ |
| Gums bleed when brushing/eating | ☐ | ☐ | ☐ | ☐ |
| White or red spots in the oral cavity | ☐ | ☐ | ☐ | ☐ |
| Ulcers or wounds | ☐ | ☐ | ☐ | ☐ |
| Previous diagnosis of oral candidiasis | ☐ | ☐ | ☐ | ☐ |
| White film on the tongue/cheeks | ☐ | ☐ | ☐ | ☐ |
| Periodontal problem diagnosticate | ☐ | ☐ | ☐ | ☐ |
| Complexes related to teeth or prostheses | ☐ | ☐ | ☐ | ☐ |
| Difficulties in daily activities due to oral problems | ☐ | ☐ | ☐ | ☐ |

**5. Psychological impact of menopausal symptoms**
**(*HADS, PHQ-9, GAD-7, MENQOL*)**
**Scale: 0 = Never | 1 = Rare | 2 = Sometimes | 3 = Frequent | 4 = Almost Daily**

| **Symptom felt** | **0** | **1** | **2** | **3** | **4** |
|---|---|---|---|---|---|
| I feel tense, nervous, or agitated | ☐ | ☐ | ☐ | ☐ | ☐ |
| I have pessimistic thoughts or feel hopeless | ☐ | ☐ | ☐ | ☐ | ☐ |
| I feel sad or demoralized | ☐ | ☐ | ☐ | ☐ | ☐ |
| I lose interest in activities that I enjoyed | ☐ | ☐ | ☐ | ☐ | ☐ |
| I have difficulty concentrating | ☐ | ☐ | ☐ | ☐ | ☐ |
| I feel irritable or "easy to provoke" | ☐ | ☐ | ☐ | ☐ | ☐ |
| I feel mentally tired, without energy | ☐ | ☐ | ☐ | ☐ | ☐ |
| I have difficulty falling asleep or wake up often for no reason | ☐ | ☐ | ☐ | ☐ | ☐ |
| I feel worthless or that I no longer have the same confidence in myself | ☐ | ☐ | ☐ | ☐ | ☐ |

**Additional questions (single check):**

1. Do you feel that menopause has affected your self-esteem (femininity, social role)?
☐ Not At All ☐ Little ☐ Moderate ☐ Much ☐ Very Much

2. Have you ever felt isolated or misunderstood about the symptoms you experienced?
☐ Never ☐ Rarely ☐ Sometimes ☐ Frequently ☐ Always

3. Did you feel the need for psychological support (professional or family)?
☐ No
☐ Yes, but I didn't seek support
☐ Yes, and I received support
