# Supplementary material for: Development and Initial Psychometric Validation of the Multidimensional Menopausal Burden Questionnaire (MMBQ-44) in a Sample of Romanian Women
Source: Diagnostics (Basel). 2026 Aug 14;16(16):2574. doi: 10.3390/diagnostics16162574 (PMC13512511; doi:10.3390/diagnostics16162574)
Supplement: Supplementary file 1 [file diagnostics-16-02574-s001.zip › Supplementary Tables.pdf]

Supplementary Table S1. Item-level descriptive statistics for all 44 items of the MMBQ-44 (N = 308).

Domain	Item	Mean	SD	Skew	Kurt	% Floor	% Ceiling
MRS	Hot flashes or night sweats	1.33	1.24	0.66	-0.57	32.1	7.5
	Difficulty sleeping due to hot flashes	1.07	1.31	1.05	-0.08	47.4	9.4
	Anxiety or nervousness	1.51	1.26	0.56	-0.71	24.4	10.1
	Feeling depressed	0.92	1.21	1.21	0.39	52.9	5.8
	Mood swings	1.19	1.16	0.81	-0.20	33.8	5.2
	Fatigue or exhaustion	1.77	1.27	0.34	-0.95	16.6	13.0
	Decrease in physical strength	1.55	1.17	0.49	-0.62	19.2	7.1
	Lack of energy	1.56	1.16	0.45	-0.62	18.8	7.1
	Joint or muscle pain	1.67	1.28	0.26	-1.04	22.7	9.4
	Bloating / abdominal discomfort / gallbladder attacks	1.46	1.25	0.53	-0.70	26.9	8.4
	Weight gain	1.87	1.38	0.18	-1.20	19.8	17.5
	Headache (migraine, headache)	1.11	1.21	0.90	-0.20	40.6	5.5
	Frequent urination	1.12	1.17	0.83	-0.25	39.3	4.5
	Urinary incontinence (when coughing or without coughing)	0.86	1.20	1.33	0.66	55.5	5.5
	Dizziness, vertigo, or balance problems	0.94	1.09	1.05	0.30	45.1	2.9
	Decreased interest in sexual activity	1.61	1.35	0.40	-1.00	26.6	13.0
	Vaginal dryness, itching, burning	1.09	1.23	1.00	-0.01	42.2	6.8
	Discomfort/pain during intercourse	0.91	1.18	1.30	0.80	50.3	6.2
XI	Feeling of "dry mouth"	0.68	0.78	0.96	0.28	49.4	2.6
	Difficulty swallowing dry food	0.40	0.65	1.57	2.11	67.5	1.0
	The need to sip liquids in order to swallow	0.28	0.59	2.30	5.27	78.6	1.0
	Speech difficulties	0.30	0.59	2.10	4.50	75.6	1.0
	Dry, cracked lips	0.53	0.74	1.43	1.82	58.8	2.9
	Nocturnal thirst	0.64	0.79	1.22	1.07	52.3	3.9
	Changes in smell	0.50	0.81	1.53	1.43	66.6	3.2
	Food tastes different than it used to be	0.28	0.58	2.08	3.65	78.6	0.3
OHIP	Dental problems causing difficulty pronouncing letters/words	0.28	0.58	2.13	4.33	77.6	0.6

	Gums are sore when brushing/eating	0.60	0.77	1.22	1.02	54.9	2.9
	White or red spots in the oral cavity	0.19	0.47	2.77	8.11	84.7	0.3
	Mouth ulcers or sores	0.14	0.43	3.39	12.44	88.6	0.3
	History of oral candidiasis	0.15	0.47	3.36	11.29	89.3	0.3
	White coating on the tongue or cheeks	0.24	0.54	2.43	6.17	80.5	0.6
	Diagnosed periodontal disease	0.47	0.80	1.71	2.10	69.2	3.9
	Complexes related to teeth or dentures	0.46	0.78	1.73	2.33	68.2	3.6
	Difficulty in daily activities due to oral problems	0.19	0.49	2.97	9.59	85.1	0.6
**DOGS**	I feel tense, nervous, or agitated	1.52	1.13	0.58	-0.30	18.2	7.5
	I have pessimistic thoughts or feel hopeless	0.86	1.11	1.26	0.75	51.0	3.9
	I feel sad or demoralized	0.97	1.08	1.23	0.98	40.3	4.5
	I lose interest in activities that I used to enjoy	0.87	1.02	1.24	1.02	44.5	2.6
	I have difficulty concentrating	1.11	1.13	1.04	0.42	34.1	5.5
	I feel irritable or "easily provoked"	1.34	1.13	0.80	-0.03	23.4	6.5
	I feel mentally tired, without energy	1.47	1.25	0.63	-0.62	24.4	9.7
	I have difficulty falling asleep or I wake up often for no reason	1.49	1.36	0.58	-0.87	29.2	13.0
	I feel worthless or have lost self-confidence	0.88	1.13	1.27	0.80	50.3	4.5

Skew and kurtosis are Fisher-corrected; values |skew| > 1 or |kurtosis| > 2 indicate marked non-normality, expected for Likert items in low-prevalence symptom domains. % Floor / % Ceiling at item level are reported on the native item Likert scale (0–4 for MRS and PSY items; 0–3 for XI and OHIP items).

Supplementary Table S2. Pattern matrix loadings for all 44 items on the four extracted factors.

Domain	Item	F1	F2	F3	F4	Best F	Best λ
**MRS**	Hot flashes or night sweats	-0.04	0.04	0.21	0.77	F4	0.77
	Difficulty sleeping due to	-0.03	0.02	0.24	0.78		0.78

	hot flashes						
	Anxiety or nervousness	0.57	-0.18	0.24	*0.31*		0.57
	Feeling depressed	0.79	-0.12	0.04	0.09	F1	0.79
	Mood swings	0.64	-0.07	0.17	0.20		0.64
	Fatigue or exhaustion	0.41	-0.16	0.55	0.11		0.55
	Decrease in physical strength	0.27	-0.14	0.67	0.07		0.67
	Lack of energy	*0.35*	-0.21	0.62	0.11		0.62
	Joint or muscle pain	0.00	-0.14	0.72	0.16		0.72
	Bloating / abdominal discomfort / gallbladder attacks	-0.05	-0.23	0.72	0.21		0.72
	Weight gain	0.09	-0.07	0.60	0.18	F3	0.60
	Headache (migraine, headache)	0.14	0.01	0.40	0.19		0.40
	Frequent urination	-0.19	0.20	0.65	0.26		0.65
	Urinary incontinence (when coughing or without coughing)	-0.06	0.14	0.50	0.14		0.50
	Dizziness, vertigo, or balance problems	0.01	0.01	0.62	0.18		0.62
	Decreased interest in sexual activity	0.26	0.21	0.16	*0.32*	F4	0.32
	Vaginal dryness, itching, burning	*0.40*	0.29	-0.16	*0.38*	F1	0.40
	Discomfort/pain during intercourse	0.46	0.22	-0.23	*0.35*		0.46
	Feeling of "dry mouth"	0.03	0.23	0.45	0.13		0.45
	Difficulty swallowing dry food	-0.07	0.27	0.54	-0.10		0.54
	The need to sip liquids in order to swallow	-0.10	*0.34*	0.47	-0.19	F3	0.47
**XI**	Speech difficulties	0.08	0.05	0.55	-0.23		0.55
	Dry, cracked lips	0.14	0.16	0.41	-0.23		0.41
	Nocturnal thirst	-0.00	*0.39*	0.21	0.16	F2	0.39
	Changes in smell	0.05	0.29	0.44	-0.28		0.44
	Food tastes different than it used to be	0.10	0.29	*0.35*	-0.27	F3	0.35
	Dental problems causing difficulty pronouncing letters/words	0.13	0.41	0.15	-0.06		0.41
	Gums are sore when brushing/eating	0.08	0.51	0.10	-0.04		0.51
**OHIP**	White or red spots in the oral cavity	-0.12	0.76	0.01	0.13	F2	0.76
	Mouth ulcers or sores	-0.05	0.57	0.01	-0.01		0.57
	History of oral candidiasis	-0.11	0.77	-0.10	0.18		0.77
	White coating on the	-0.28	0.62	0.14	0.16		0.62

	tongue or cheeks						
	Diagnosed periodontal disease	0.16	0.56	-0.12	-0.11		0.56
	Complexes related to teeth or dentures	0.15	0.67	-0.13	0.03		0.67
	Difficulty in daily activities due to oral problems	0.08	0.80	-0.20	0.09		0.80
**DOGS**	I feel tense, nervous, or agitated	0.72	-0.04	0.17	0.03	F1	0.72
	I have pessimistic thoughts or feel hopeless	0.97	0.00	-0.17	-0.06		0.97
	I feel sad or demoralized	0.99	0.09	-0.20	-0.06		0.99
	I lose interest in activities that I used to enjoy	0.79	0.02	0.07	-0.08		0.79
	I have difficulty concentrating	0.70	0.07	0.16	-0.14		0.70
	I feel irritable or "easily provoked"	0.74	-0.05	0.18	-0.05		0.74
	I feel mentally tired, without energy	0.73	-0.12	*0.33*	-0.10		0.73
	I have difficulty falling asleep or I wake up often for no reason	0.48	0.02	0.21	0.21		0.48
	I feel worthless or have lost self-confidence	0.84	0.07	-0.03	-0.10		0.84

Supplementary Table S3

Mean and standard deviation of the continuous characteristics reported in Table 1, by menopausal stage (N = 308). Provided in response to Reviewer 2, comment 13. Table 1 of the main text reports the median and interquartile range, because the Shapiro–Wilk test rejected normality for every one of these variables and the between-stage comparisons were performed with the Kruskal–Wallis test; the means are given here for readers who prefer a familiar summary.

**Variable**	**Total (N = 308)**	**Perimenopause (n = 66)**	**Menopause (n = 40)**	**Postmenopause (n = 202)**	**Shapiro–Wilk p**
Age (years)	53.4 ± 5.5	48.8 ± 2.5	48.7 ± 2.9	55.8 ± 5.1	6.2×10^{-8}
Height (cm)	164.1 ± 6.4	165.5 ± 6.6	164.3 ± 5.5	163.7 ± 6.4	9.2×10^{-3}
Weight (kg)	73.5 ± 14.0	70.2 ± 12.9	70.9 ± 13.2	75.1 ± 14.4	5.6×10^{-6}
BMI (kg/m^2)	27.3 ± 5.3	25.6 ± 4.3	26.4 ± 5.2	28.1 ± 5.5	9.6×10^{-8}
Years since last menstruation	4.6 ± 4.0	0.0 ± 0.0	1.0 ± 0.0	6.9 ± 3.2	1.0×10^{-13}

Values are mean ± standard deviation. Shapiro–Wilk p-values are for the total sample; all reject normality at $\alpha = 0.05$. BMI, body mass index.

Supplementary Table S4

Domain coverage of menopause-specific, oral-health and psychological instruments in current use, compared with the MMBQ-44. Provided in response to the Academic Editor, comment 4.

Instrument	Vasomotor / somatic	Uro-genital	Psychological	Oral / oral-sensory	Menopause-specific
Menopause Rating Scale (MRS)	Yes	Yes	Partial	No	Yes
Menopause-Specific Quality of Life (MENQOL)	Yes	Yes	Partial	No	Yes
Greene Climacteric Scale	Yes	Partial	Yes	No	Yes
Xerostomia Inventory (XI)	No	No	No	Yes (dryness only)	No
Oral Health Impact Profile-14 (OHIP-14)	No	No	Partial (social impact)	Yes (functional impact)	No
PHQ-9 / GAD-7 / HADS	No	No	Yes	No	No
MMBQ-44 (present study)	Yes	Yes	Yes	Yes	Yes

Menopause-specific instruments assess vasomotor, somatic, urogenital and, in part, psychological symptoms, but none evaluates oral or oral-sensory burden. Oral-health instruments evaluate dry-mouth symptoms and oral-health-related quality of life, but are not menopause-specific and omit the vasomotor, urogenital and psychological domains. Generic psychological screening instruments assess affective symptoms in isolation from the physical domains. The MMBQ-44 is the only instrument in this comparison that covers all four domains within a single administration and on a common 0–100 scoring metric.
